# Lead poisoning in a 10-year-old boy with cyclic vomiting as the first symptom: a case report

**DOI:** 10.3389/fped.2025.1580368

**Published:** 2025-08-06

**Authors:** Liping Ding, Liyuan Wang, Zhiling Wang

**Affiliations:** ^1^ Department of Pediatrics, West China Second University Hospital, Sichuan University, Chengdu, Sichuan, China; ^2^ Key Laboratory of Birth Defects and Related Diseases of Women and Children (Sichuan University), Ministry of Education, Chengdu, Sichuan, China

**Keywords:** lead poisoning, cyclic vomiting, cyclic vomiting syndrome, neurological involvement, pediatric

## Abstract

**Introduction:**

Lead is a naturally occurring metal with highly toxic effects on humans, particularly children, who are particularly vulnerable to its long-lasting adverse impacts.

**Patient concerns:**

This report presents the case of a 10-year-old boy with a 10-month history of recurrent vomiting. No organic lesions were identified. However, the symptoms were inconsistent with cyclic vomiting syndrome (CVS), given the presence of growth retardation, vitamin D deficiency, bilateral cervical lymphadenopathy, academic difficulties, and impaired concentration.

**Diagnosis:**

Detailed history-taking revealed proximity to a lead mining facility. Blood lead level (BLL) was significantly elevated (382 μg/L), confirming chronic lead poisoning.

**Interventions:**

Chelation therapy with dimercaptosuccinic acid (DMSA) was administered for 19 days at a 350 mg/m^2^ dose per administration.

**Outcomes:**

Following treatment, BLL decreased to 168 µg/L, accompanied by significant improvement in vomiting and abdominal pain.

**Conclusion:**

Pediatric lead poisoning should be considered in the differential diagnosis of children presenting with cyclic vomiting and neurological symptoms, due to its diagnostic complexity.

## Introduction

Lead poisoning remains a significant global public health concern due to its potential for irreversible damage in children. Clinical manifestations include acute encephalopathy, peripheral neuropathy, hearing loss, neurobehavioral dysfunction, abdominal pain, constipation, growth retardation, vitamin D deficiency, anemia, and kidney disease (1). However, cyclic vomiting as an initial symptom is rarely reported in pediatric cases. This study describes a child with lead poisoning presenting primarily with cyclic vomiting; the clinical features were retrospectively analyzed. This case provides valuable insights into the association between lead exposure and atypical gastrointestinal symptoms in children.

## Case presentation

A 10-year-old boy was admitted to our hospital with a 10-month history of recurrent vomiting. Episodes occurred without obvious triggers. Although non-projectile, the vomiting was severe, occurring more than 20 times per day and lasting 3–7 days, followed by symptom-free intervals of approximately 20 days. As episodes progressed, bilious and coffee-ground emesis developed. During severe episodes, the child experienced lethargy and epigastric pain, without radiation or referred pain. Notably, the child had no symptoms of cough, bloating, diarrhea, constipation, or hematochezia. Between episodes, his mental status and appetite returned to normal after appropriate treatment.

On admission, a comprehensive physical examination was performed. The child appeared alert but undernourished. His height (130 cm) and weight (24 kg) were 1–2 standard deviations below the mean for his age. No skin rash was observed. However, multiple enlarged, soft, mobile, and non-tender lymph nodes were palpable bilaterally, with the largest measuring approximately 3.0 cm × 1.5 cm. The tonsils were grade II enlarged without purulent discharge. Abdominal examination revealed epigastric tenderness without rebound pain or guarding. The liver and spleen were not palpable, and bowel sounds were normal. Cardiopulmonary and neurological examinations were unremarkable.

Gastroscopy at a local hospital revealed chronic non-atrophic gastritis with bile reflux. Upon admission to our hospital, routine blood, stool, and urine tests showed no significant abnormalities. Liver and kidney function, serum electrolytes, blood ammonia, and β-hydroxybutyrate were all within normal limits (Table 1). However, serum vitamin D was deficient at 5.9 ng/ml (deficiency < 10 ng/ml). Thyroid function tests revealed mildly reduced T3, T4, and free T3 levels. Cervical ultrasonography showed enlarged bilateral lymphadenopathy, with the largest nodes measuring 3.3 cm × 1.0 cm on the left and 3.4 cm × 1.0 cm on the right. Non-contrast abdominal computed tomography (CT) revealed no abnormalities.

**Table 1:** Laboratory results.

Test	Value	Reference range	Test	Value	Reference range
WBC (10^9^/L)	10.0	3.6–9.7	ALT(U/L)	24	21–72
RBC (10^12^/L)	5.03	3.82–5.5	AST(U/L)	33	17–59
Hb (g/L)	123	110–146	Cr(µmol/L)	30	35.9–83.1
PLT (10^9^/L)	243	100–450	Vit D(ng/ml）	5.9	Deficiency <10
AMON (µmol/L)	<9	9–30	T3(nmol/L)	1.36	1.4–3.7
BHB (mmol/L)	0.5	0–0.27	T4(nmol/L)	81.4	82–171
P (mmol/L)	1.72	1.45–2.10	FT3(pmol/L)	4.71	5.1–10.1
Ca (mmol/L)	2.22	2.10–2.55	ALP(U/L)	200	45–125

WBC, white blood cell count; RBC, red blood cell count; Hb, hemoglobin; PLT, platelet count; AMON, blood ammonia; BHB, β-hydroxybutyrate; P, Phosphorus; Ca, Calcium; ALT, Alanine aminotransferase; AST, Aspartate aminotransferase; Cr, Creatinine; Vit D, Vitamin D; T3, Triiodothyronine; T4, Thyroxine; FT3, free triiodothyronine; ALP, alkaline phosphatase.

Due to the absence of conclusive findings, a detailed environmental history was obtained. It was discovered that a lead mining facility had been established near the child's residence within the past two years. Considering his academic difficulties and impaired concentration, lead poisoning was suspected. Blood lead level (BLL) testing at an occupational disease hospital revealed a substantially elevated level of 382 μg/L (reference range: 0–100 μg/L), confirming a diagnosis of moderate lead poisoning. Chelation therapy with dimercaptosuccinic acid (DMSA) was initiated. For the first 5 days, DMSA was administered three times daily at a dose of 1,050 mg/m^2^/ day. This regimen was followed by 14 days of twice-daily dosing (700 mg/m^2^/day), totaling 19 days of treatment. Following therapy, BLL decreased to 168 μg/L (Table 2), significantly improving vomiting and abdominal pain. At one-year follow-up, after closure of the nearby lead mine, the child remained symptom-free, with no recurrence of vomiting, abdominal pain, or fatigue.

**Table 2:** Blood lead level (BLL) trends during treatment.

Date	BLL (µg/L)
2020-01-13	382
2020-01-15	316
2020-01-19	239
2020-01-23	204
2020-01-31	168

## Discussion

In this case, the child's clinical presentation demonstrated cyclical vomiting patterns with symptom-free intervals. Based on physical examination and ancillary test results, common organic causes of vomiting were initially excluded. Among functional gastrointestinal disorders, cyclic vomiting syndrome (CVS) was first considered—a condition characterized by recurrent episodes of intense vomiting, with or without retching, occurring in a stereotypical pattern and followed by return to baseline health between episodes (2). Stress and infections are well-established triggers of CVS; additional factors such as certain foods (such as chocolate, cheese, and caffeine), fasting, fever, sleep deprivation, allergies, diet, and menstruation may precipitate episodes (3, 4).

CVS represents a final common manifestation of diverse pathophysiological mechanisms, rather than a single etiology (5). Its pathogenesis has been linked to gut–brain axis disturbances, mitochondrial enzyme dysfunction, gastrointestinal motility disorders, calcium channel abnormalities, and hypothalamic–pituitary–adrenal (HPA) axis hyperactivity (6). Among these, HPA axis overactivation is hypothesized to affect autonomic regulation (specifically sympathetic stimulation and vagal inhibition), leading to altered intestinal motility. Maternal lead exposure has been associated with long-term changes in HPA axis function, including hypercortisolism (7). Although the mechanisms remain unclear, lead may directly affect HPA axis regulation or exert indirect effects through neurochemical disruption, potentially triggering cyclic vomiting.

However, CVS alone could not fully account for the patient's other clinical findings—namely, vitamin D deficiency, growth retardation, and bilateral cervical lymphadenopathy. This discrepancy prompted a more detailed environmental history, revealing the presence of a lead mine plant near his residence. A considerably elevated BLL confirmed moderate lead poisoning. Although the child had no history of pica, he frequently played near the lead mine, and the hand-to-mouth behavior likely contributed to ingesting lead-contaminated dust or soil. The resolution of symptoms following chelation therapy strongly supports a causal link between lead exposure and his cyclic vomiting episodes. Lead, a naturally occurring heavy metal, is pervasive in the environment—found in air, dust, soil, and water—and widely used in mining, construction, paint, gasoline, ceramics, food, and traditional Chinese medicine (8). As a potential neurotoxin, lead causes irreversible harm in children, making lead poisoning a significant public health concern worldwide (9). Both the Centers for Disease Control and Prevention (CDC) and the World Health Organization (WHO) recognize elevated blood lead levels in children as a critical marker of increased health risk (10, 11).

Lead poisoning presents in two distinct forms based on exposure duration. Acute poisoning results primarily from brief exposure to high lead concentrations, with chronic lead poisoning—more prevalent in children over 2–3 years of age—typically manifesting 3–6 months following continuous environmental exposure. The 10-month disease duration in this case aligns with a diagnosis of chronic lead poisoning. Neurological manifestations dominate in chronic exposure, especially concerning impaired cognitive development. As observed in this patient, potential outcomes include reduced intelligence quotient (IQ), attention deficits, antisocial behavioral tendencies, and poor academic performance (11). To the best of our knowledge, this represents the first reported case of chronic lead poisoning presenting with cyclic vomiting as the primary and predominant symptom. Initially, both lead poisoning and CVS were considered as differential diagnoses. In adults, CVS may coexist with various conditions, such as marijuana smoking, migraine, mental illness, diabetes, and irritable bowel syndrome. Besides managing cyclic vomiting episodes, treating coexisting conditions is also crucial. This strategy alleviates symptoms and leads to complete resolution within 1–2 years of treatment (12). While this conclusion is primarily based on adult data, it may also apply to children. However, in this case, the child's vomiting improved significantly following lead chelation therapy alone and did not recur during a 1-year follow-up without lead exposure. Hence, it is conclusive that lead poisoning was the primary cause of his cyclic vomiting, an unusual and distinctive feature of this case.

Several reports support our findings. One study described a worker in a lead-manganese smelter who experienced nausea, vomiting, abdominal colic, dizziness, headache, and memory loss for over a year. Although intermittent abdominal pain was noted, the periodicity of vomiting episodes remained unspecified (13). Another investigation (unpublished) identified an association between persistent gastrointestinal symptoms and BLL ≥500 μg/L. Interestingly, the prevalence of intermittent gastrointestinal symptoms was twice as high in children with BLLs >200 μg/L compared to those with lower levels (14). In our case, the child's BLL was 382 μg/L—within the 200–500 μg/L range—and his recurrent symptoms aligned with previous observation.

The long-term consequences of lead poisoning in children encompass growth retardation, vitamin D deficiency, anemia, and renal impairment (15, 16). Although this child had no signs of anemia or nephropathy, he exhibited growth retardation and vitamin D deficiency. No skeletal deformities were noted, and serum calcium and phosphorus levels were normal; however, alkaline phosphatase (ALP) was mildly elevated (Table 1). His vitamin D deficiency was likely multifactorial, due to both chronic lead exposure and limited ultraviolet B radiation in a geographically sun-restricted basin (17, 18). Kersey et al. (19) found a negative but statistically insignificant association between BLLs and serum 25-hydroxyvitamin D [25(OH)D] levels in healthy toddlers and children under age 6. Lead may impair hepatic 25-hydroxylase activity, the enzyme responsible for converting vitamin D to its main circulating form, 25(OH)D. To address the deficiency, a two-phase supplementation regimen was implemented: an initial intensive phase of cholecalciferol (2,000 IU/day for 12 weeks), followed by a maintenance phase (600 IU/day) (18). Cervical lymphadenopathy and tonsillar hypertrophy were observed. Children residing in urban or industrialized areas are reported to have higher tonsillar and lymph node enlargement rates, possibly due to increased colonization of pathogenic bacteria on the tonsils' surface (20).

Given the irreversible neurocognitive and behavioral effects of lead exposure in children, primary prevention is paramount (9, 10). Recent studies indicate that children residing near lead mines, smelters, and chemical plants are at particularly high risk. These industries may leak substantial amounts of lead during production, contaminating regional air, soil, and water. The density of lead mining and lead smelting and chemical companies was positively correlated with the BLLs of children, higher BLLs indicated a more significant density of lead smelting and chemical companies in a specific area. The greater the volume and duration of lead leakage to a local area, the greater its effect on the BLLs of local children (21). Therefore, lead mining and smelting enterprises face significant challenges in strictly controlling emissions and enhancing environmental surveillance. Simultaneously, residential development in adjacent areas must be prohibited to prevent continued lead exposure among children. Furthermore, in confirmed cases of lead poisoning, children must remain in lead-free environments following chelation therapy to prevent re-exposure (1).

However, despite our repeated recommendations during the one-year telephone follow-up, the child's parents refused re-evaluation of the child's BLL. Remote mountainous residence, inadequate transportation, financial constraints, and the absence of recurring clinical symptoms over the past year influenced their decision. Consequently, we were unable to further assess whether BLL rebound indeed occurred in this case. Future research should focus on monitoring dynamic BLL changes following chelation therapy.

## Conclusions

To the best of our knowledge, this is the first report of cyclic vomiting caused by lead poisoning. Given the declining incidence and atypical manifestations, lead poisoning is frequently misdiagnosed. This clinical case may be a valuable reference for future clinical diagnosis and treatment. Lead poisoning should be considered in pediatric patients presenting with cyclic vomiting and neurological symptoms.

## Data Availability

The original contributions presented in the study are included in the article/Supplementary Material, further inquiries can be directed to the corresponding author.
